# Persistent Drug-Induced Parkinsonism in Patients with Normal Dopamine Transporter Imaging

**DOI:** 10.1371/journal.pone.0157410

**Published:** 2016-06-13

**Authors:** Jin Yong Hong, Mun Kyung Sunwoo, Jungsu S. Oh, Jae Seung Kim, Young H. Sohn, Phil Hyu Lee

**Affiliations:** 1 Department of Neurology, Yonsei University Wonju College of Medicine, Wonju, Korea; 2 Department of Neurology, Bundang Jesaeng General Hospital, Seongnam, Korea; 3 Department of Nuclear Medicine, Asan Medical Center, University of Ulsan College of Medicine, Seoul, Korea; 4 Department of Neurology and Brain Research Institute, Yonsei University College of Medicine, Seoul, Korea; 5 Severance Biomedical Science Institute, Yonsei University College of Medicine, Seoul, Korea; University of Nebraska Medical center, UNITED STATES

## Abstract

Functional neuroimaging for the dopamine transporter (DAT) is used to distinguish drug-induced parkinsonism (DIP) from subclinical Parkinson’s disease (PD). Although DIP patients who show a normal DAT image are expected to recover completely, some do not. We investigated whether these patients showed changes in striatal DAT activity using semi-quantitative analysis of ^18^F-FP-CIT PET data. DIP patients with visually normal DAT images were selected from medical records. The subjects were classified as patients who recovered partially (PR) or completely within 12 months (CR). The ^18^F-FP-CIT uptake in each striatal subregion was compared between the CR and the PR groups. In total, 41 and 9 patients of the CR and PR groups were assessed, respectively. The two patient groups were comparable in terms of clinical characteristics including age, sex, and severity of parkinsonism. From semi-quantitative analysis of the PET image, the PR patients showed a relatively lower ligand uptake in the ventral striatum, the anterior putamen and the posterior putamen compared with the CR patients. This result suggests that persistent DIP in patients with visually normal DAT imaging may be associated with subtle decrement of DAT activity.

## Introduction

Drug-induced parkinsonism (DIP) is commonly seen in movement disorder clinics [1, 2]. Although the parkinsonian symptoms develop after administration of certain drugs, there is heterogeneity in the nigrostriatal status among patients with DIP. Up to 43% of them show normal activity of nigrostriatal neurons, suggesting that their parkinsonism is caused solely by the offending drugs (pure DIP) [3–5]. The remainder shows impaired activity of nigrostriatal neurons, hence their parkinsonism may develop by unmasking the preclinical stage of Parkinson's disease (PD) by the offending drugs (unmasked PD). However, the characteristics of parkinsonian symptoms are insufficient to distinguish pure DIP from unmasked PD [6]. Another report showed that asymmetric parkinsonism is more prevalent in unmasked PD, but a third of patients with pure DIP also had asymmetry [7]. For this reason, functional imaging techniques to assess the nigrostriatal presynaptic status, especially that using ligands of the dopamine transporter (DAT), are used to distinguish pure DIP from unmasked PD [6–13].

In addition, several early reports showed that patients developed PD after complete remission of DIP (antedated PD) [14–16]. A recent study reported two patients whose parkinsonism recurred within 2 years of full remission, and their DAT activities were nearly normal initially but were decreased at the time of the follow-up scan [13]. In contrast, pathological studies revealed neuronal loss of substantia nigra and Lewy bodies in the completely recovered patients with DIP [17, 18]. Taken together, it seems that gap exists between the clinical symptoms and pathologic or imaging findings.

Interestingly, some DIP patients who have normal DAT activity show persistent parkinsonism after the cessation of the offending drug [10, 11, 19]. Although the DAT imaging of these patients looks normal, the persistent symptoms may imply permanent damage in the dopaminergic pathway. Recently, a population-based elderly cohort study showed that the risk of PD was increased by 3.2-fold after exposure to neuroleptics [20]. This result also implies that DIP is a risk factor for progressive dopaminergic degeneration.

In this study, we investigated whether there is any change in DAT activity in partially recovered DIP patients who show normal DAT imaging using semi-quantitative analysis of ^18^F-FP-CIT PET data.

## Methods

### Subjects

We reviewed the medical records from a movement disorder clinic of a tertiary referral center, and selected the DIP patients. DIP was diagnosed according to a previously proposed criteria as follows [21]: (1) the presence of two or more cardinal symptoms of parkinsonism, (2) an absence of parkinsonian symptoms before exposure to the offending drug, (3) a disappearance or significant improvement in parkinsonism after withdrawal of the offending drug, (4) no better explanation for the parkinsonism. To rule out patients with unmasked PD or vascular parkinsonism, those showing abnormal findings in either brain MRI or ^18^F-FP-CIT PET scans were excluded. After semi-quantitative analysis of the DAT density, we excluded the patients whose DAT density in any subregion was lower than 2 standard deviations (SD) below the mean of the normal data [12]. The normal DAT density value was obtained from 68 healthy controls (age 66.5 ± 7.4 years, male/female 25/43) who had been administered ^18^F-FP-CIT PET for a medical check-up. The normal DAT data of the controls are shown in Table 1.

**Table 1:** Normal data of ^18^F-FP-CIT uptake. Mean value and standard deviation (SD) of each striatal subregions were from 68 healthy controls. Patient who showed uptake lower than mean-2SD in any subregion was excluded from this study.

	Caudate	Ventral striatum	Whole putamen	Anterior putamen	Posterior putamen
Mean	2.53	3.12	3.43	3.75	3.23
SD	0.65	0.64	0.69	0.81	0.63
Mean-2SD	1.23	1.84	2.05	2.13	1.97

SD: standard deviation

The patients were divided according to their degree of recovery. The patients whose parkinsonian symptoms did not recover completely within 12 months after discontinuing the offending drug were classified as the "partial recovery (PR)" group, and those who recovered completely from DIP were classified as the "complete recovery (CR)" group. The patients with a follow-up duration shorter than 12 months without full recovery were excluded.

### Ethics statement

The study protocol was approved by the institutional review board (IRB) on human experimentation, and was exempt from providing informed consent by the IRB due to the retrospective design. All information of patients was anonymized prior to the analyses.

### Acquisition of ^18^F-FP-CIT PET data

The ^18^F-FP-CIT PET scans were performed using a GE PET-CT DSTe scanner (GE Discovery STE, GE Healthcare Technologies, Milwaukee, WI, USA), which obtains images with a three-dimensional resolution of 2.3 mm full width at half-maximum. All subjects fasted for at least 6 hours before the PET scan. After fasting, 5 mCi (185 MBq) of ^18^F-FP-CIT was injected intravenously, and the images were acquired in the three-dimensional mode at 120 KVp and 380 mAs during a 20-minutes session, performed at 90 minutes after injection.

### Quantitative analysis of ^18^F-FP-CIT PET data

Quantitative analyses were performed following a modified version of a previously described procedure [22, 23]. Image processing was performed using SPM8 (Wellcome Department of Imaging Neuroscience, Institute of Neurology, UCL, London, UK) with Matlab 2013a for Windows (Math Works, Natick, MA, USA). Quantitative analyses were based on volumes of interest (VOIs), which were defined based on a template in standard space. To remove inter-subject anatomical variability, all reconstructed PET images were spatially normalized to the Montreal Neurology Institute (MNI) template space using a standard ^18^F-FP-CIT PET template which was generated using ^18^F-FP-CIT PET and T1 MR images from 13 normal controls. Eight VOIs of bilateral striatal subregions and one occipital VOI were drawn on a co-registered spatially normalized single T1 MR and ^18^F-FP-CIT PET template image on MRIcro version 1.37 (Chris Rorden, Columbia, SC, USA), based on a previous study [22]. The striatum was divided into the caudate, the ventral striatum, the anterior putamen, and the posterior putamen. The VOI for the ventral striatum was defined according to previously defined criteria [24], and the boundary between the anterior and the posterior putamen was the anterior commissure coronal plane. The outer boundaries of the striatal subregions were visually determined by the characteristic dense grey signal of the striatum, which readily distinguished these subregions from adjacent structures. These VOIs were adjusted by a minor translation in our in-house editing software called ANTIQUE [25]. DAT activity was calculated by the non-displaceable binding potential (BP_ND_), which was defined as (mean standardized uptake value of the striatal subregions VOI–mean standardized uptake value of the occipital VOI)/mean standardized uptake value of the occipital VOI [26].

### Statistical analyses

The Mann-Whitney U test and chi-square test were used to compare demographic characteristics between the PR and CR groups. The Mann-Whitney U test was also used to compare the DAT activity of each striatal subregion between the groups. Statistical analyses and plotting were performed using IBM SPSS Statistics 21 (IBM, Armonk, NY, USA), and *p* < 0.05 was considered statistically significant.

## Results

### Study subjects and demographic data

A total of 52 DIP patients who showed a visually normal DAT density on ^18^F-FP-CIT PET were selected from historical medical record. However, two patients who had a DAT density slightly below the cut-off were excluded. Among the remaining 50 patients, nine had recovered partially (PR group) from parkinsonism, and the other 41 had recovered completely (CR group).

The demographic characteristics of the subjects are shown in Table 2, and the original data are presented in S1 File. There was no significant difference in sex distribution or age at the time of the PET scan between the groups. The PR and CR groups were similar in age at onset of parkinsonian symptoms, the time from onset of parkinsonism to PET scan, and UPDRS motor scores. The composition of the offending drugs was also comparable.

**Table 2:** Demographic characteristics of the subjects.

		PR (n = 9)	CR (n = 41)	P value
Male/female, n		5/4	10/31	0.106^a^
Age at PET scan, yr		70.7 (66.0–77.0)	71.5 (67.8–77.0)	1.000^b^
Age at onset of parkinsonism, yr		69.9 (64.9–75.9)	70.3 (66.7–75.5)	0.882^b^
Time from onset to PET scan, yr		1.0 (0.4–1.6)	0.4 (0.2–1.4)	0.921^b^
UPDRS motor score		27 (20–36)	27 (20–33)	0.637^b^
Offending drugs				0.878^a^
	Antiemetic	5 (56%)	18 (44%)	
	Flunarizine	2 (22%)	7 (17%)	
	Diltiazem	0 (0%)	2 (5%)	
	Valproate	0 (0%)	2 (5%)	
	Antipsychotic	1 (11%)	2 (5%)	
	SSRI	0 (0%)	2 (5%)	
	Two or more	1 (11%)	8 (20%)	

Data are expressed as median (interquartile range) or n (%).

PR: patients who recovered partially; CR: patients who recovered completely, PET: Positron emission tomography; UPDRS: Unified Parkinson’s disease rating scale; N/A: not applicable.

^a^ Chi-square test

^b^ Mann-Whitney U test

The clinical information of the PR patients is presented in Table 3. Parkinsonism occurred between the ages of 53 and 83 years in five men and four women. The patients visited the hospital 1 to 26 months after symptom onset and were followed up for 12 to 60 months after cessation of the offending drugs. The UPDRS motor scores varied between 19 and 56 points at initial examination but improved to 6–10 points at follow-up.

**Table 3:** Clinical information for partially recovered patients with drug-induced parkinsonism.

Patients	sex	Age of onset (yr)	Starting offender–onset (mo)	Onset—visit hospital (mo)	Initial symptoms						Follow-up symptoms						Follow-up (mo)	Offending drug
					UPDRS III	RT	PT	R	BK	PI	UPDRS III	RT	PT	R	BK	PI		
1	M	65	10	12	20	-/-	++/++	++/++	+/+	+	10	-/-	+/+	+/+	+/+	-	60	Amitriptyline, mirtazapine
2	M	65	6	3	40	-/-	++/+++	++/++	+++/+++	+	7	-/-	+/-	+/-	+/-	-	27	Flunarizine
3	M	70	4	9	32	-/+	+/++	++/++	++/++	-	7	-/-	-/-	-/+	-/+	-	26	Flunarizine
4	M	75	2	1	25	-/++	+/+	-/+	++/++	-	9	-/-	-/+	-/+	-/+	-	41	Levosulpiride
5	M	83	2	1	27	-/-	-/-	++/+++	++/+++	+	9	-/-	-/-	-/+	-/+	-	38	Levosulpiride
6	F	53	1	12	56	++/+	+++/+++	+++/++	+++/+++	+	9	+/-	+/-	+/-	+/-	-	26	Metoclopramide
7	F	67	24	26	20	+/+	++/+	+/+	++/+	+	10	-/-	+/+	+/+	+/+	-	57	Perphenazine
8	F	75	1	2	32	-/-	+/+	-/+	+/++	++	10	-/-	+/+	-/+	-/+	-	42	Levosulpiride
9	F	77	12	24	19	+/-	+/+	+/-	+/+	-	6	-/-	+/-	+/-	+/-	-	12	Levosulpiride

RT: rest tremor; PT: postural tremor; R: rigidity; BK: bradykinesia; PI: postural instability

### DAT density on ^18^F-FP-CIT PET

**Fig 1:**
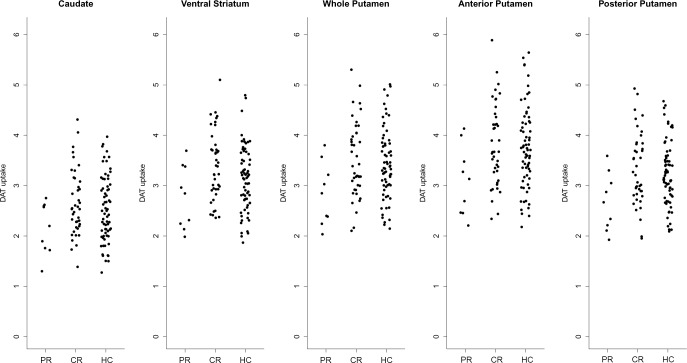
The ^18^F-FP-CIT uptake in the striatal subregions of partially (PR) and completely recovered (CR) patients and healthy controls (HC).

The ^18^F-FP-CIT uptake in the striatal subregions in the two groups are shown in Table 4. S1 File, and Fig 1. The PR patients showed a lower DAT density in the ventral striatum and putamen compared with the CR patients. In the putamen, there were significant differences in both the anterior and posterior putamen. The DAT density of the caudate was comparable between the PR and the CR patients.

**Table 4:** The ^18^F-FP-CIT uptake in striatal subregions.

		PR	CR	P value
Caudate		2.20 (1.74–2.61)	2.54 (2.18–3.11)	0.069
Ventral striatum		2.85 (2.19–3.39)	3.38 (2.92–3.76)	0.018
Putamen		2.85 (2.31–3.39)	3.39 (2.96–4.05)	0.016
	Anterior	3.13 (2.46–3.74)	3.67 (3.19–4.39)	0.018
	Posterior	2.67 (2.16–3.18)	3.21 (2.82–3.83)	0.017

Data are expressed as median (interquartile range).

PR: patients who recovered partially; CR: patients who recovered completely

## Discussion

In the present study, the PR patients with DIP showed a relatively lower DAT density in putamen and ventral striatum compared with the CR patients, even though all patients of both groups had normal DAT density.

Persistent parkinsonism in these patients seems to be surprising, but similar cases have already been observed in previous studies. In the first study using PET in patients with DIP, Burn and Brooks presented nine patients whose DAT imaging was normal [11]. Among them, five did not recover completely after more than 6 months of drug washout. The major remnant symptom was postural tremor, but two patients showed a rest tremor, and one showed bradykinesia. Olivares Romero et al. also presented two patients, among 15 with normal ^123^I-FP-CIT SPECT images, who did not fully recover during the 14 or 15 months of follow-up [10]. However, almost none of the other studies assessed the recovery of parkinsonism in DIP patients with normal DAT images. Other studies classified the partially recovered patients as PD [3] and either excluded them from study [27] or did not follow-up until the completion of recovery [6, 7, 9, 12].

The parkinsonian symptoms of partially recovered patients did not aggravate again during follow-up period. Previous reports described that the parkinsonian symptoms of antedated PD developed within 2.5 years after DIP remission [13–16]. While, for eight of nine patients of this study who recovered partially from DIP showed stable parkinsonism for more than 2 years, their clinical courses seem to be different from that of antedated or unmasked classical PD. Therefore, we can speculate that the parkinsonism was due to permanent dopaminergic dysfunction induced or unmasking very mild and stable PD pathology by offending drugs.

Absent parkinsonian symptom before administration of offending drugs and persistent parkinsonism after drug discontinuation imply permanent presynaptic dysfunction or the loss of presynaptic dopaminergic neurons. The pharmacological dysfunction of the D2 receptor by the offending drug is the widely accepted pathophysiology of DIP, whereas there are several studies suggesting a neurotoxic effect of D2 receptor blocking agents on dopaminergic neurons. An experimental study demonstrated that a pyridinium metabolite derived from haloperidol (HPP+) had a neurotoxic effect on dopaminergic and serotonergic neurons [28]. Another animal study showed that chronic administration of haloperidol resulted in oxidative damage and decreased turnover of dopamine and norepinephrine [29], and that tyrosine hydroxylase (TH) activity of the substantia nigra was persistently down-regulated in rats treated for 8 weeks with haloperidol, despite a recovery in the TH-positive cell count [30]. In a clinical study, elevated levels of oxidative markers were observed in the CSF of patients with tardive dyskinesia [31]. Moreover, not only a reduced TH level but also neurodegeneration was observed in the ventral pallidum of haloperidol- or eticlopride-treated rats [32]. These results support that exposure to dopamine receptor blocking agents may cause biochemical or pathological changes in the dopaminergic system of the human brain, even though there is no direct evidence of neurodegeneration by offending drugs in patients with DIP.

Despite the clinical course of partially recovered patients, possible underlying pathology cannot be ruled out. Two autopsy studies found neuronal loss of substantia nigra and Lewy bodies in DIP patients whose parkinsonism disappeared completely. The researchers postulated that mild PD pathology may underlie even pure DIP, and this suggestion accords with our results. However, there is no pathological report on patients showing both persistent parkinsonism and normal range of DAT activity until now, hence further autopsy studies to examine underlying pathology are warranted.

Semi-quantitative analysis revealed that the ^18^F-FP-CIT uptake in PR patients was reduced to 86.0–89.4% of that in the CR patients. The motor symptoms of PD appear when there is 48–68% nigral dopaminergic cell loss [33]; therefore, this reduction in the rate of ^18^F-FP-CIT uptake in PR patients seems to be insufficient to develop clinical symptoms. However, previous PET studies on parkinsonism patients with frontotemporal dementia (FTD) or Alzheimer's disease (AD) demonstrated only a 20% reduction in ligand uptake compared with healthy controls [34, 35]. Such a relatively low rate of reduction may imply concomitant damage of the postsynaptic neurons in neurodegenerative disease presenting parkinsonism. A pathological study found that the dopaminergic and dopaminoreceptive neurons were decreased equally in patients with FTD and parkinsonism linked to chromosome 17 (FTDP-17) [36]; another pathological study showed that AD patients with parkinsonism had significant pre- and postsynaptic neuronal loss when compared with those without parkinsonism [37]. Similarly, in terms of DIP, if the pharmacological blocking effect of the offending drug also becomes neurotoxic, then relatively lower amount of presynaptic neuronal loss may be sufficient to present symptoms of parkinsonism. To confirm this hypothesis, further studies exploring postsynaptic activity in DIP patients having both normal DAT images and persistent parkinsonism are needed.

Several previous studies have demonstrated that the visual interpretation of DAT imaging is useful to differentiate pure DIP from unmasked PD [3, 9–11]. Moreover, recent studies have distinguished pure DIP from unmasked PD using cut-off values of 1–2.5 SD below the mean derived from normal data [3, 12]. We also recruited DIP patients with normal appearing DAT images and ruled out two DIP patients among them who showed a binding potential slightly lower than 2 SD below the mean from normal data. This process may prevent inclusion of patients with very early-stage PD among the study subjects.

The present study had some limitations. Although longitudinal data were used, its retrospective design was insufficient for drawing clear conclusions. Second, the duration of follow-up of the subjects was relatively short for determining whether the parkinsonism of the PR patients was progressive. Third, it is unclear whether there were permanent postsynaptic changes or any relationship between the presynaptic and postsynaptic environment. Also, PET data do not exactly reflect the neuronal status. Pathological studies are required to evaluate the implications of the present results.

In conclusion, we report relatively lower DAT activity in patients who have not recovered completely from DIP, despite normal appearing DAT images, compared with those who recovered completely. This result suggests that persistent DIP in patients with visually normal DAT imaging may be associated with subtle decrement of DAT activity.

## Supporting Information

S1 File The dataset of this study. (XLSX)
